# Electroconvulsive therapy and adiposity-related parameters in treatment-resistant depressed patients

**DOI:** 10.1007/s00702-022-02475-8

**Published:** 2022-02-25

**Authors:** Hannah Benedictine Maier, Christoph Pollak, Nicole Moschny, Sermin Toto, Colin Schlatt, Christian K. Eberlein, Wolfgang Sperling, Johannes Kornhuber, Kai G. Kahl, Stefan Bleich, Alexandra Neyazi, Helge Frieling

**Affiliations:** 1 Department of Psychiatry, Social Psychiatry and Psychotherapy, Hannover Medical School, Carl-Neuberg-Str. 1, 30625 Hannover, Germany; 2 Department of Psychiatry and Psychotherapy, Friedrich-Alexander-University Erlangen-Nuremberg, Erlangen, Germany

**Keywords:** ECT, Glucose, Insulin, HOMA, Serum lipids, HDL, LDL, Triglyceride, BMI, Depression

## Abstract

Obesity is often accompanied by major depressive disorder (MDD), and vice versa. Latest research findings suggest the body mass index (BMI) to play a role in antidepressant treatment response in general. Our study aims to examine whether adiposity-related parameters such as BMI, glucose homeostasis, or serum lipids are associated with remission to electroconvulsive therapy (ECT). A pilot study (PS, *n* = 9) and a glucose study (GS, *n* = 29) were conducted. Blood was withdrawn directly before and 15 min (GS) as well as 1 h (PS) after the first ECT and directly before the last one (usually an ECT series comprised up to twelve sessions). BMI was associated with remission in the PS (remitters: *M* = 28, SD = 2.5; non-remitters: *M* = 22, SD = 2.08; *t*(7) = 3.325, *p* < 0.001, *d* = 0.24) but not in the GS or when pooled together. Glucose and insulin levels increased significantly after a single ECT session (GS: glucose: *F* (2,25.66) = 39.04, *p* < 0.001; insulin: PS: *F* (2,83) = 25.8, *p* < 0.001; GS: *F* (2,25.87) = 3.97, *p* < 0.05) but no chronic effect was detectable. Serum lipids were neither significantly altered after a single ECT session nor during a whole course of ECT. There was no difference between remitters and non-remitters in insulin, glucose, or serum lipid levels. Our study is lacking the differentiation between abdominal and peripheral fat distribution, and the sample size is small. Unexpectedly, BMI, glucose homeostasis, and lipid serum levels did not differ in patients remitting during ECT. In contrast to recently published studies, we cannot confirm the hypothesis that BMI may have an impact on ECT response.

## Introduction

Obesity and major depressive disorder (MDD) are both devastating, co-occurring diseases that reduce the quality of life of affected patients (Mannan et al. 2016; Nigatu et al. 2016). Furthermore both diseases increase the risk of developing type 2 diabetes, cardiovascular diseases, metabolic syndrome, or a dysregulated lipid metabolism (Penninx and Lange 2018; Shin et al. 2008). Irregularities in lipids, e.g., hypertriglyceridemia, might be a result of a hyperactive hypothalamic–pituitary–adrenal (HPA) axis that is frequently found in a subtype of depressed subjects (Penninx and Lange 2018). In a recent meta-analysis, lower serum total cholesterol and LDL (low-density lipoprotein) levels were associated with suicide attempts in patients suffering from MDD (Li et al. 2020). Wagner and colleagues found serum lipid levels to be associated with depression severity and depression per se (Wagner et al. 2019). Besides, a population-based cohort study found evidence of weight gain and irregularities in glucose and insulin to be partially attributable to antidepressant drugs (Gafoor et al. 2018).Additionally, p-mGPCR antagonists (such as quetiapine) bear a relative high risk of changing insulin sensitivity or glucose levels, even without causing weight gain or adiposity (Kowalchuk et al. 2019). Those effects might be mediated by the central nervous system (a known target of antidepressants and p-mGPCR antagonists), as the parasympathicus and sympathicus innervate multiple organs relevant for glucose homeostasis. To be more precise, there is evidence that the dopaminergic receptor 2 (D_2_) might be involved in developing insulin resistance through the activation of sympathetic nerve fibers and a direct effect on pancreatic beta-cells (Kowalchuk et al. 2019).

Patients receiving electroconvulsive therapy (ECT)—one of the most powerful treatment options for treatment-resistant MDD—are often more severely ill compared to patients taking pharmaceuticals only. These patients are more likely to receive psychotropic drugs with weight gain as an adverse effect (e.g., lithium, mirtazapine, valproic acid, or quetiapine) (Kellner et al. 2015). In addition to the effect of medication, depression itself is associated with obesity (Mannan et al. 2016); in part due to chronic stress, which is present in a proportion of depressed patients. The aforementioned hyperactive HPA axis can also contribute to an increase in body weight. Recent studies reported overweight patients to experience a superior response to antidepressant treatment than non-overweight ones, particularly in the case of, e.g., pharmacotherapy (Dreimüller et al. 2019; Puzhko et al. 2020), including ketamine (Freeman et al. 2020), or ECT (Moss and Vaidya 2006). The current study asks whether BMI or levels of adiposity-associated parameters (such as lipids, glucose, and insulin) are affected by ECT or related to treatment outcome. Studies examining the possible effects of ECT on lipids, glucose, and insulin are sparse, and if present, often conducted without keeping the patient’s particular treatment response in mind. To give some examples, Rasmussen and Ryan (2005) found a significant rise of glucose after a single ECT session in 30 patients (out of 33) but measured neither its chronic effect nor its relation to remission (Rasmussen and Ryan 2005). Regarding ECT’s long-term impact, Williams et al. (1992) reported the glucose response to stay consistent but the amplitude of insulin increase to diminish during a five-treatment session. Interestingly, the decrease in insulin response was associated with a poor outcome (Williams et al. 1992). In diabetic patients, conflicting data are available concerning the insulin requirements necessary while undergoing ECT (Netzel et al. 2002; Reddy and Novler 1996; Normand and Jenike 1984; Fakhri et al. 1980; Yudofsky and Rosenthal 1980). In this context, Rasmussen and colleagues found the rise of glucose to be comparable between diabetic and non-diabetic patients (Rasmussen et al.^ 2006^), whereas a case report of a 73-year-old woman, who had a 20-years history of insulin-dependent diabetes mellitus, suggested lower requirements of insulin after a whole course of ECT (Normand and Jenike 1984). In a retrospective study of 19 insulin-dependent diabetic patients, Netzel and colleagues did not find a significant difference in insulin requirements before and after the ECT series (Netzel et al. 2002). Regarding lipids, changes in serum lipids were found after a course of ECT (Aksay et al. 2016; Ghanizadeh et al.^ 2012^; Kurt et al. 2007), but none of the previous studies analyzed serum lipids in terms of outcome to ECT.

Taken together, there is evidence that BMI, glucose homeostasis, and serum lipids play a role in depression or at least in a subgroup thereof. Their association with remission is yet to be elucidated. To answer our research question, we first studied a small group of patients who received ECT. Further on, we wanted to verify our results by analyzing an independent, larger and better characterized cohort. We set out to verify the hypothesis that BMI and remission to ECT are associated. Additionally, our study aimed to shed light on the acute and chronic effects of glucose homeostasis and peripheral serum lipid levels during a course of ECT in remitters and non-remitters.

## Methods

### Patients and study design

Both studies adhered to the Declaration of Helsinki (1964) and its later amendments. The pilot study was approved by the Ethics Committee of the University of Erlangen (3252–2006), the Northern German Electroconvulsive Therapy Outcome Registry (Norddeutsches Elektrokonvulsionstherapie Outcome Register [NEKTOR]) by the Ethics Committee of Hannover Medical School (2842–2015). Prior to study inclusion and after the procedures had been fully explained to the participating patients, written informed consent was obtained. Non-responsiveness to two state-of-the-art antidepressant treatment trials, at least, was defined as pharmacoresistance in either study (Berlim and Turecki 2007). Remission was defined as a MADRS score ≤ 10.

### Pilot study

The 1st cohort included nine pharmacoresistant depressed patients (five females, four males). All patients were recruited from an inpatient population at the Department of Psychiatry and Psychotherapy of the University Hospital Erlangen-Nuremberg. MDD was diagnosed according to the first version of the Structured Clinical Interview for Diagnostic (SKID) and Statistical Manual of Mental Disorders, Fourth Edition (DSM IV). Depression severity was assessed before the first and the last ECT using the German versions of the Montgomery–Åsberg Depression Rating Scale (MADRS) and Beck Depression Inventory (BDI-II).

### Glucose study

The 2nd cohort (*n* = 29; 20 females, 9 males) was an analysis from NEKTOR. The cohort was acquired at the Department of Psychiatry, Social Psychiatry and Psychotherapy at Hannover Medical School. Outcome analysis was performed for patients with MDD according to the ICD-10 (International Statistical Classification of Diseases and Related Health Problems 10th Revision). Depression severity was assessed using BDI-II and MADRS. For comorbid axis-II-disorders, the Structured Clinical Interview for Diagnostic II (SKID-II) and DSM IV were used.

### Administration of ECT

ECT was administered three times per week usually for the duration of 4 weeks unless ECT was ended due to adverse events or the patient’s wish. The Thymatron® IV brief-pulse device (Somatics, LLC) was used. Usually, the ECT series were started with right unilateral electrode placement (RUL) and adjusted to bilateral stimulation due to non-responsiveness and ECT seizure quality at the sixth ECT session, if needed. For stimulation intensity, the age method was used as commonly practiced in the facility. Anesthesia was obtained using methohexital or propofol, remifentanil, and succinylcholine or mivacurium. An electroencephalogram was obtained using two channels placed frontomastoidal that was monitored during ECT to document seizure duration. To control motor seizure activity, a blood pressure cuff was inflated on one extremity—usually on the right lower leg—before muscle relaxation. Study details for the pilot study have already been reported elsewhere (Kleimann et al. 2015; Maier et al. 2018; Müller et al. 2012).

### Blood samples

#### Pilot study

Blood samples were taken directly before the first, 1 h after the first, and directly prior to the last ECT sessions. The blood samples were immediately stored at − 80 °C after collection, centrifugation and aliquoting for further processing. Insulin and C-peptide levels were measured using an electrochemiluminescence immunoassay (ECLIA; Cobas 8000, Modul e801; Fa. Roche).

#### Glucose study

Blood was withdrawn directly before and 15 min after the first and directly prior the last ECT. 2 K EDTA-Gel and Serum S-Monovettes® (Sarstedt AG & Co, Nümbrecht, Germany) were used as collection tubes. The tubes were temporally stored at 4 °C up to 3 h and at room temperature (RT; 1 h, Serum S-Monovettes® only). Blood was centrifuged (2000x*g*, 10 min, RT: 2 K EDTA-Gel Monovettes®, 4 °C: Serum S-Monovettes®), aliquoted and kept at − 80 °C until further use. Glucose levels were assessed using an enzymatic reference method with hexokinase (Cobas 8000, module c701; Fa. Roche). Insulin levels were measured using an ECLIA (Cobas 8000, module e801; Fa. Roche) and cholesterol and triglyceride levels were analyzed using an enzymatic color test (Cobas 8000, module c701, Fa. Roche).

##### Homeostasis model assessment (HOMA)

The homeostasis model assessment (HOMA) is a mathematical assessment to quantify beta-cell function and insulin resistance in patients from fasting levels of glucose and insulin. The model takes the balance between insulin secretion from fasting of glucose, hepatic glucose output and insulin into account (Matthews et al. 1985). HOMA-IR (HOMA of insulin resistance) describes an index of basal glucose and insulin levels divided by 22.5. HOMA-B (HOMA of beta-cell function) consists of the product of basal insulin levels and 20 divided by the basal glucose concentration minus 3.5 (Matthews et al. 1985; Wallace et al. 2004). HOMA has been widely validated and has proven to be of significant value in evaluating the diabetes risk in individuals (Song et al. 2007).

### Statistical analysis

Fisher’s exact test and Mann–Whitney *U* test were used for the baseline analysis of the demographics (body mass index, age, gender), psychometric (BDI, MADRS) and other clinical baseline parameters between remitters and non-remitters to ECT (e.g., anxiety disorders, personality disorders). Spearman’s Rho test was performed for baseline psychometric parameters, and serum levels. For group comparison, Student’s *t* test was used. The analysis of insulin, glucose, triglyceride, HDL or LDL cholesterol and remission/changes over time under ECT were performed using mixed linear models (pilot study: fixed effects and their interactions: ECT number, measurement number, and remission; random factors: age and gender; glucose study: fixed effects and their interactions: timepoint and remission). Bonferroni correction was used for multiple comparisons in mixed linear models. The results are presented as mean (*M*) ± standard deviation (SD) or ± standard error (SE). *P* values of less than 0.05 (two tailed) were considered significant. For analysis of data, IBM SPSS Statistics for Windows, Version 26.0 (IBM Corp.), was used. For data presentation, Graph Pad Prism 5 (Graph Pad Inc.) and R 3.6.1 were used.

## Results

### Patients’ baseline demographic characteristics

The pooled baseline characteristics of the pilot and glucose study are shown in Table 1. There were no significant differences between remitters and non-remitters except for the use of first-generation antipsychotics as well as the comorbidities anxiety and personality disorder (both Fisher’s exact test: *p* < 0.05) in the glucose study. For two patients–both remitters–there was a lack of previous medical history. Age and gender did not show any association with the measured parameters and were thus not included as covariates.

**Table 1:** Baseline demographic characteristics of remitters and non-remitters

Characteristics	Pilot study + Glucose study			Pilot study			Glucose study		
	Whole cohort (*n* = 38)	Non-remitters (*n* = 26)	Remitters (*n* = 14)	Whole cohort (*n* = 9)	Non-remitters (*n* = 6)	Remitters (*n* = 3)	Whole cohort (*n* = 29)	Non-remitters (*n* = 19)	Remitters (*n* = 10)
Age, years, mean (SD)	52 (± 16)	52 (± 16)	53 (± 15)	45 (± 15)	49 (± 16)	37 (± 11)	54 (± 16)	50 (± 16)	58 (± 13)
Gender, *n* (%)									
Female	25 (65.8)	17 (68.0)	8 (61.5)	5 (55.6)	3 (50)	2 (66.7)	20 (69)	12 (60.6)	6 (60)
Male	13 (34.2)	8 (32.0)	5 (38.5)	4 (44.4)	3 (50)	1 (33.3)	9 (31)	5 (29.4)	4 (40)
MADRS, mean (SD)	32.6 (± 8.5)	33 (± 8)	32 (± 10)	32 (± 8)	34 (± 6)	28 (± 13)	33 (± 9)	32 (± 8)	34 (± 10)
BDI, mean (SD)	35.63 (± 11.05)	38 (± 11)	31 (± 11)	35 (± 11)	39 (± 8)	28 (± 14)	36 (± 11)	38 (± 12)	32 (± 11)
Duration of current depressive episode, weeks, mean (SD)	–	–	–	–	–	–	28 (± 20)	34 (± 21)	14 (± 11)
Age at initial diagnosis, years, mean (SD)	31.93 (± 15)	34.0 (± 15)	27.0 (± 14)	32 (± 14)	39 (± 12)	19 (± 7)	32 (± 16)	31 (± 17)	31 (± 16)
Suicidality, *n* (%)	–	–	–	–	–	–	9 (23.7)	6 (31.6)	3 (30)
Pharmacoresistance, *n* (%)	27 (71.1)	17 (68)	10 (90.9)	9 (100)	6 (100)	3 (100)	18 (66.7)	10 (58.8)	7 (87.5)
History of Psychotherapy, *n* (%)	–	–	–	–	–	–	19 (50)	13 (76.5)	6 (66.7)
First-time ECT, *n* (%)	–	–	–	–	–	–	21 (72)	15 (78.9)	6 (60)
Psychotic features (glucose study only)	–	–	–	–	–	–	8 (21.1)	5 (26.3)	3 (30)
Bipolar Disorder (glucose study only)	–	–	–	–	–	–	4 (10.5)	1 (5.3)	3 (30)
*Medication*									
Antidepressants, *n* (%)	–	–	–	–	–	–	17 (44.7)	11 (57.9)	6 (60)
Typical antipsychotics, *n* (%)	–	–	–	–	–	–	7 (18.4)	2 (10.5) *	5 (50)*
Atypical antipsychotics, *n* (%)	–	–	–	–	–	–	13 (34.2)	9 (47.4)	4 (40)
Lithium, *n* (%)	–	–	–	–	–	–	1 (2.6)	0	1 (10)
Benzodiazepines, *n* (%)	–	–	–	–	–	–	13 (34.2)	7 (36.8)	6 (60)
*Psychiatric comorbidities*									
Anxiety disorder, *n* (%)	–	–	–	–	–	–	8 (21.1)	8 (42.1) *	0 (0) *
Personality disorder, *n* (%)	–	–	–	–	–	–	6 (15.8)	6 (31.6) *	0 (0) *
Addiction disease, *n* (%)	–	–	–	–	–	–	8 (21.1)	6 (31.6)	2 (20)

Fisher’s exact test and Mann–Whitney U test revealed no significant differences between remitters and non-remitters in the respective groups (all *p* > 0.100) except for typical antipsychotics, anxiety, and personality disorder (*p < 0.05). *n* number, *SD* standard deviation, *MADRS* Montgomery–Åsberg Depression Rating Scale, *BDI-II* Beck Depression Inventory, *ECT* Electroconvulsive therapy

We calculated the homeostatic model assessment (HOMA) to quantify beta-cell function (HOMA beta) and insulin resistance (HOMA IR) (Matthews et al. 1985) only in the glucose study. There were no differences in the baseline HOMA IR or HOMA beta between remitters and non-remitters (Mann–Whitney *U*: HOMA IR: *p* = n.s.; HOMA beta: *p* = n.s.; Table 2).

### BMI does not differ between remitters and non-remitters to ECT

**Fig. 1:**
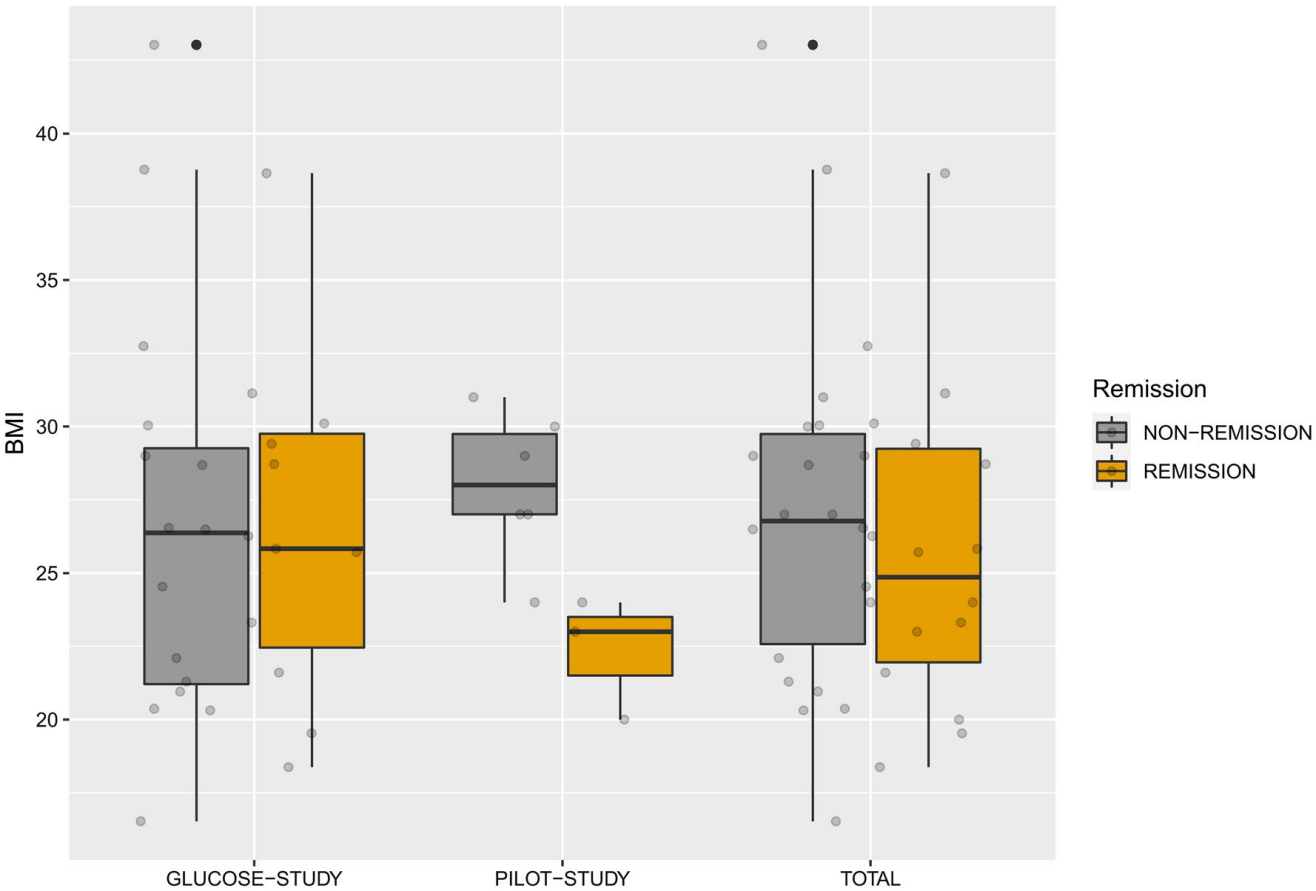
Body mass index (BMI) and remission. In the pilot study, remitters (*n* = 3) had a significantly higher baseline BMI compared to non-remitters (*n* = 6). In the glucose study and when data were pooled, there was no significant difference concerning the baseline BMI in remitters (*n* = 10 and *n* = 13, respectively) versus non-remitters (*n* = 19 and *n* = 25, respectively)

In the pilot study, remitters had a significantly higher baseline BMI (*M* = 28, SD = 2,5) compared to non-remitters (*M* = 22, SD = 2.08; *t* (7) = 3.325, *p* < 0.001, Cohen’s *d* = 0.24). In the glucose study and when data were pooled, there was no significant difference concerning the baseline BMI in remitters (*M* = 26, SD = 5.93 and *M* = 25, SD = 5.48, respectively) versus non-remitters (*M* = 27, SD = 6.82, *t* (25) = 0.503 and *M* = 27.4, SD = 5.91, *t* (34) = 1.152, respectively; both *p* = n.s.; Fig. 1).

### Insulin and C-peptide alter in a single ECT session in the pilot study

In the pilot study, mixed linear models revealed a significant association of ECT treatment with insulin and C-peptide when measured before and 1 h after a single ECT session (insulin: *F* (2,83) = 25.8, *p* < 0.001, C-peptide: *F* (2,84) = 30.09, *p* < 0.001) as well as when compared between remitters and non-remitters (insulin: *F* (2,83) = 7.59, *p* < 0.05; C-peptide: *F* (2,84) = 12.14, *p* = 0.001). There was additionally a significant increase in insulin during the ECT series (before first and before last ECT: *F* (1,15) = 5.63, *p* < 0.05) but no change in C-peptide was detectable (*F* (1,15) = 4.38, *p* = n.s.).

**Table 2:** Baseline metabolic characteristics of remitters and non-remitters

Characteristics	Pilot study + Glucose study			Pilot study			Glucose study		
	Whole cohort (*n* = 38)	Non-remitters (*n* = 26)	Remitters (*n* = 14)	Whole cohort (*n* = 9)	Non-remitters(*n* = 6)	Remitters (*n* = 3)	Whole cohort (*n* = 29)	Non-remitters (*n* = 19)	Remitters (*n* = 10)
Diabetes mellitus, *n* (%)	11 (28.9)	7 (28)	4 (30.8)	8 (88.9)	5 (83.3)	3 (100)	3 (10.3)	2 (11.8)	1 (10)
Arterial hypertension, *n* (%)	–	–	–	–	–	–	12 (41.4)	7 (36.8)	5 (50)
Hyperlipoproteinemia, *n* (%)	8 (21.1)	4 (16)	4 (30.8)	5 (55.6)	2 (33.3)	3 (100)	3 (10.3)	2 (11.8)	1 (10)
Not obese or overweight (BMI < 25), n (%)	14 (41.2)	7 (33.3)	7 (53.8)	4 (44.4)	1 (16.7)	3 (100)	10 (40)	5 (35.7)	4 (40)
Overweight (BMI 25–29), *n* (%)	11 (32.4)	8 (28.1)	3 (23.1)	3 (33.3)	3 (50)	0 (0)	8 (32)	5 (35.7)	3 (30)
Obese (BMI > 30), *n* (%)	9 (26.5)	6 (28.6)	3 (23.1)	2 (22.2)	2 (33.3)	0 (0)	7 (28)	4 (28.6)	3 (30)
Glucose, mmol/l, mean (SD)	4.8 (± 0.8)	4.8 (± 0.6)	5 (± 1.1)	–	–	–	4.8 (± .8)	4,8 (± 0.6)	5.0 (± .1)
Insulin, µg/ml, mean (SD)	–	–	–	40.2 (± 32.6)	37.2 (± 33.1)	46.2 (± 38)	–	–	–
Insulin, mU/l, mean (SD)	–	–	–	–	–	–	10.3 (± 7)	10.8 (± 7.6)	9.4 (± .7)
HOMA beta, mean (SD)	–	–	–	–	–	–	174.34 (± 116.48)	168.16 (± 94.23)	175.77 (± 160.29)
HOMA IR, mean (SD)	–	–	–	–	–	–	2.35 (± 2.02)	2.43 (± 1.9)	2.3 (± 2.45)
*Insulin resistance according to HOMA IR*									
Normal (HOMA IR < 2), *n* (%)	–	–	–	–	–	–	8 (42.1)	4 (33.3)	3 (60.0)
Insulin resistance possible (HOMA IR 2–2.5), *n* (%)	–	–	–	–	–	–	3 (15.8)	3 (25)	0 (0)
Insulin resistance most likely (HOMA IR 2.5–5), *n* (%)	–	–	–	–	–	–	6 (31.6)	4 (33.3)	1 (20)
Average in type 2 diabetes mellitus (HOMA IR > 5), *n* (%)	–	–	–	–	–	–	2 (10.5)	1 (8.3)	1 (20)

Fisher’s exact test and Mann–Whitney *U* test revealed no significant differences between remitters and non-remitters in the respective groups (all *p* > 0.100)

### ECT has an acute effect on glucose, insulin, HOMA IR and triglycerides in the glucose study

**Fig. 2:**
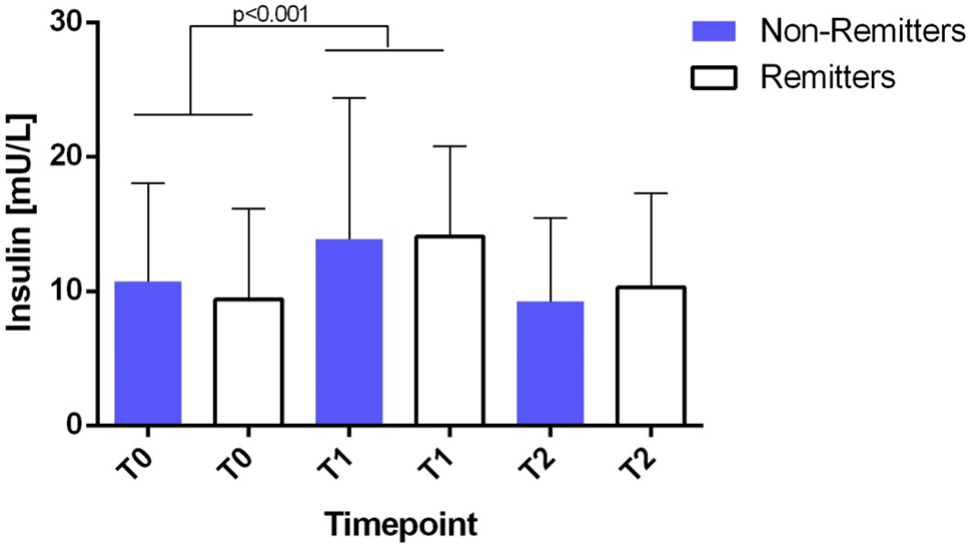
Insulin serum levels during a course of ECT in the glucose study. Figure shows levels of insulin during a course of ECT in the glucose study. We found a significant increase from T0 to T1. No difference was shown when remitters (*n* = 10) and non-remitters (*n* = 19) were compared. Error bars show the standard error of the mean (SEM). T0 = Baseline, T1 = 15 min after first ECT, T2 = directly prior to last ECT

**Fig. 3:**
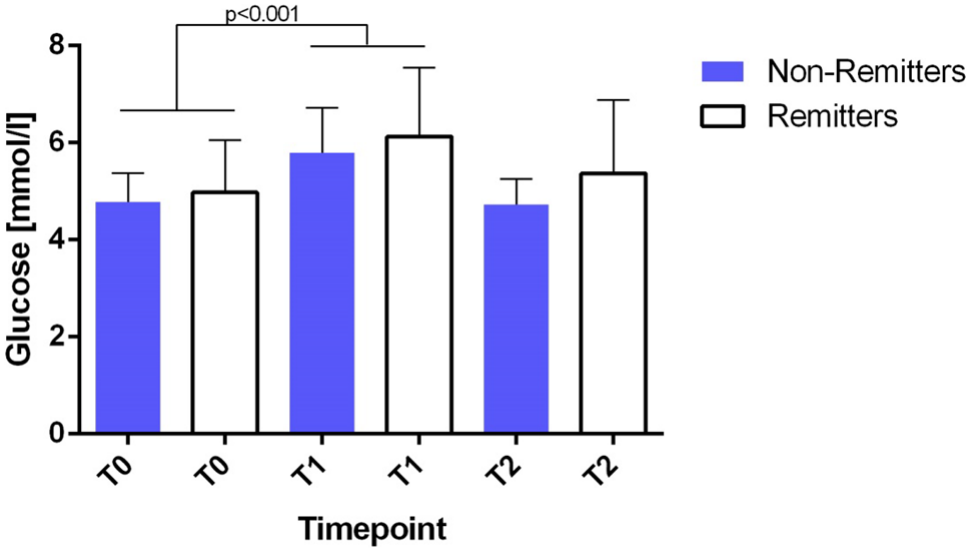
Glucose serum levels during a course of ECT in the glucose study. Figure shows levels of glucose during a course of ECT in the glucose study. There was a significant increase in glucose levels at T1. No difference was shown when remitters (*n* = 10) and non-remitters (*n* = 19) were compared. Error bars show the standard error of the mean (SEM). T0 = Baseline, T1 = 15 min after first ECT, T2 = directly prior to last ECT

We set out to replicate our findings concerning insulin. Mixed linear models revealed in the glucose study a significant acute effect of ECT (before and 15 min after ECT) on insulin (*F* (2,25.87) = 3.97, *p* < 0.05) and glucose (*F* (2,25.66) = 39.04, *p* < 0.001) serum levels. There was no significant change during the ECT series (before first and before last ECT: insulin: *F* (1, 10.19) = 9.95, *p* = n.s., Fig. 2; glucose: *F* (1, 26) = 2.00, *p* = n.s.; Fig. 3). There was no difference between remitters and non-remitters in neither insulin nor glucose serum levels (insulin: *F* (1, 21.8) = 0.13, *p* = n.s.; glucose: *F* (1, 27.9) = 2.96, *p* = n.s.). Baseline BDI positively correlated with the chronic (before 1st/before last ECT) insulin change (*r* = 0.404, *p* < 0.05).

HOMA IR showed an acute effect on a single ECT (*F* (2,28.04) = 13.14, *p* = 0.005). A chronic effect (*F* (1, 19.69) = 10.07, *p* = n.s.) or a difference between remitters and non-remitters over a course of ECT was not detectable (*F* (1, 27.28) = 1.64, *p* = n.s.). HOMA beta presented no difference in terms of an acute (*F* (2, 21.91) = 1.93, *p* = n.s.) or chronic effect (*F* (3, 23.81) = 1.55, *p* = n.s.). Remitters and non-remitters did not differ during a course of ECT (HOMA beta: *F* (1, 28.51) = 0.8, *p* = n.s.). HOMA beta and MADRS negatively correlated with each other (*r* = − 0.454, *p* < 0.05).

Triglycerides showed a significant acute effect on ECT (*F* (2,25.6) = 4.48, *p* < 0.05), but no chronic effect (*F* (3, 25.47) = 1.4, *p* = n.s.) was measurable. HDL- and LDL-cholesterol did not display an acute or chronic change within the course of ECT (HDL: *F* (2, 25.68) = 2.87, *p* = n.s.; LDL: *F* (2, 25.92) = 0.22, *p* = n.s.). Baseline BDI and chronic triglyceride change (before 1st/before last ECT) positively correlated with each other (*r* = 0.449, *p* < 0.05).

## Discussion

In our study, we were able to detect an acute increase in insulin and glucose after a single ECT session. We could not show a chronic effect over a whole ECT session. Our findings go in line with previous reports (Rasmussen and Ryan 2005; Williams et al. 1992). The underlying cause is not elucidated yet, but one possible explanation is the reflection of neural activation during ECT (Gravenstein et al. 1965). The pancreas underlies the neural control of the right vagal nerve (Fakhri et al. 1980) and, therefore, the release of glucose and especially insulin after a single ECT session could point to the spread of the seizure throughout the brain and the vagal nerve. There are additional signs for neuroendocrine responses after a single ECT session for example in the release of thyroid-stimulating hormone or prolactin release (Deakin et al. 1983; Esel et al. 2002). Additionally, to this result, we found a positive association between depression severity and the change of insulin during an ECT series. This association could imply a normalization of the stress-induced HPA axis dysregulation in a subgroup of depressed patients during an ECT series.

Another explanation for the increase of glucose could be the seizure-induced secretion of hormones like cortisol (Yrondi et al. 2018). Subtypes of depression are associated with hypercortisolism probably due to chronic stress (Mayer et al. 2018). Moreover, an increase of cortisol and glucose can be caused by higher anxiety levels due to a release of sympathetic hormones, which could occur previous to ECT and also seizure-induced (Wong et al. 2019). Hypercortisolism is known to impact glucose metabolism through decreased pancreatic secretion of insulin and increased hepatic gluconeogenesis (Ioakim et al. 2020). Since this mechanism already takes place even in slightly higher cortisol levels than usual, cortisol disturbs the glucose metabolism (Kamba et al. 2016). Another explanation for the altered glucose homeostasis could be the multiple brief fasting periods three times a week over at least 4 weeks as for the ECT sessions the patients are not allowed to eat at least 6 h before treatment due to the risk of aspiration. In regard to intermittent fasting periods and their association with glucose homeostasis or weight loss, the studies are unfortunately inconsistent (Longo and Mattson 2014; Trepanowski et al. 2017; Varady 2016). Interestingly enough, we found a negative association between beta-cell function measured with HOMA beta and depression severity. This result could emphasize the interaction between a dysregulated HPA axis and stress in depression. Although this result is preliminary and requires further research as we considered this quite interesting.

Additionally, to the glucose homeostasis, we aimed to answer the question, if BMI and ECT remission are associated as recent research associated antidepressant treatment response with BMI. In a recent review, Puzhko and colleagues pointed out, that obesity goes in line with low-grade inflammation such as C-reactive protein (CRP) and that inflammation was linked to an altered antidepressant treatment response (Haroon et al. 2018; Puzhko et al. 2020). Antidepressant medication, ketamine, and ECT all have an anti-inflammatory impact and may unfold their effect through this way (Freeman et al. 2020; Liu et al. 2014). Another link between adiposity and treatment resistance was suggested via the alteration of the HPA axis and the neurotransmitter systems (Puzhko et al. 2020). One putative mechanism for treatment resistance and adiposity could be explained through leptin resistance as circulating leptin was positively correlated with body weight and BMI. The anti-histaminergic activity of several antidepressant treatment options, such as amitriptyline or mirtazapine, putatively contributes to the aforementioned leptin resistance through dysregulation of hypothalamic nuclei (Schilling et al. 2013). Since adipokine dysregulation is also present in patients with obesity and MDD this may impact antidepressant response to medication as well as ketamine or ECT (Freeman et al. 2020). In a recent review concerning antidepressant treatment response and excess body weight as a predictor, 91.7% of the studies reported a clinically relevant association between weight status and antidepressive response (Puzhko et al. 2020). In our study, we were solely able to find a correlation between the higher BMI and remission to ECT in the pilot study. One possible explanation could be that we did not assess the hip-to-waist ratio as Puzhko and colleagues pointed out, that anthropometric measures of obesity besides BMI and body weight, such as hip-to-waist ratio or waist circumference, are not in standard use yet. Especially the subgroup of patients having a hyperactive HPA axis may have abdominal obesity and therefore might be a subgroup responding better to antidepressive treatment. Interestingly enough, Dreimüller and colleagues performed a post hoc analysis of their Early Medication Change study for non-responders after 14 days of escitalopram where the patients were randomized to either switch to venlafaxine or continue taking escitalopram. They found overweight patients at baseline to show the best response to antidepressant treatment. Additionally, they found an association between an initial increase in BMI and a larger decrease in depression severity during follow-up (Dreimüller et al. 2019). The underlying mechanisms for these findings are not known yet.

In our study, severely depressed patients with a long psychiatric history were included. Those patients are more likely to undergo ECT and therefore may have a higher BMI and are additionally older. Older patients slightly tend to respond better to ECT than younger people (Haq et al. 2015). The better response may be referred to the higher stimulation dosage used for older patients.

In a group of treatment-resistant patients with MDD who received intravenous ketamine, Freeman and colleagues found a more robust acute antidepressant response to ketamine in patients with higher BMI or obesity (Freeman et al. 2020). One explanation could be that ketamine treatment is usually given in mg/kg based on the patient’s body weight in contrast to antidepressants (Freeman et al. 2020). The authors stated that a higher BMI or fat mass should be considered as a variable associated with the response to ketamine, even though the underlying mechanisms are still unclear (Freeman et al. 2020). In contrast to that theory, Puzhko and colleagues found an association between higher BMI and response to antidepressants (Puzhko et al. 2020). This may point towards a subgroup of depressed patients with a higher BMI maybe having stress-related depression rather than dysthymia and, therefore, respond well to antidepressant treatment. In contrast to the findings of Freeman and Puzhko, the resistance of antidepressant treatment was also associated with higher BMI. One possible explanation for the treatment resistance in patients with higher BMI could be the presence of comorbidities such as sleep apnea, metabolic syndrome, or asthma as those conditions are known to contribute to more severe depression and influence the treatment response (Chapman et al. 2005; Moussavi et al. 2007).

Not only BMI and the glucose homeostasis are important variables concerning depressed patients undergoing ECT, but also serum lipid levels as depressed individuals seem to have altered serum lipid levels when compared to healthy controls (Enko et al. 2018). Lower HDL levels were even associated with depression severity (Enko et al. 2018; Kuwano et al. 2018). In our study, we found no association between lipid levels and depression severity except BDI-II had a positive association with the triglyceride change over a whole course of ECT. Concerning the serum lipid levels and electroconvulsive therapy, Aksay and colleagues found an increase of serum lipids when measured before and after a course of ECT (Aksay et al. 2016). We were not able to replicate the results of Aksay and colleagues as the serum lipid levels did not change significantly regarding a single ECT session or during a whole ECT treatment. In contrast to our study, the time of blood withdrawing was different in the study of Aksay and colleagues where they sampled the blood before the first ECT and between 1 and 7 days after the last ECT.

One major limitation of our study is the limited sample size. There are much larger studies necessary to be able to conclude whether BMI, glucose, insulin, and lipids influences ECT outcome. Another limitation is the insufficient assessment of the metabolic syndrome as this may also have an impact on ECT success. Unfortunately, we did not assess the weight at the end of the ECT series. Since the measurement of insulin and glucose was not carried out immediately after the blood was withdrawn, values may have been falsified, e.g. insulin can be broken down by hemolysis and erythrocytes dismantle glucose.

Another limitation of our study is that we did not assess the waist-to-hip-ratio as we cannot distinguish between abdominal fat distribution and peripheral fat distribution. At least Vicennati and Pasquali found in their study a difference in aforementioned fat distributions and cortisol concentrations in response to acute stressors (Vicennati and Pasquali 2000). Additionally, according to the review by Rodriguez and colleagues the abdominally obese individuals demonstrate a greater acute stress-related cortisol response (Incollingo Rodriguez et al. 2015). Accordingly, depressed patients with an abdominal fat distribution should be considered as a different subgroup. Furthermore, that group pointed out, that it could also be possible to have a clinically “healthy” BMI but still exhibit HPA axis dysregulation due to high abdominal adiposity (Incollingo Rodriguez et al. 2015). Therefore, in future studies the waist-to-hip-ratio should be measured when assessing obesity/BMI.

In conclusion, we were not able to confirm the hypothesis that BMI, glucose homeostasis, or lipid serum levels may impact ECT response. Nevertheless, insulin and glucose are increasing after a single ECT and might display the spread of the seizure throughout the brain and the vagal nerve. For this reason, this system should be further investigated in search of an answer according to the principle of action of ECT.

## Data Availability

The dataset used during the present study is available from the corresponding author on reasonable request.
